# What are women’s experiences of seeking to plan a vaginal breech birth? A systematic review and qualitative meta-synthesis

**DOI:** 10.3310/nihropenres.13329.1

**Published:** 2023-01-20

**Authors:** Ritika Roy, Cecilia Gray, Charlene Akyiaa Prempeh-Bonsu, Shawn Walker

**Affiliations:** 1 School of Life Course Sciences, Faculty of Life Sciences & Medicine, King's College London, London, SE1 7EH, UK; 2 Independent lay member of the research team, King's College London, London, UK; 3 Women and Children's Health, King's College London, London, SE1 7EH, UK; 4 Women and Children's Services, Chelsea and Westminster Hospital NHS Foundation Trust, London, SW10 9NH, UK

**Keywords:** breech presentation, women’s experiences, systematic review, qualitative meta-synthesis, grounded theory, vaginal breech birth

## Abstract

**Background:**

Guidelines for breech management at term emphasise choice and informed decision-making. Despite this, the choice of vaginal breech birth (VBB), is not always available or accessible. We aimed to describe the experiences of women seeking a VBB as reported in primary research and to offer strategies for improving this experience that are grounded in evidence.

**Methods:**

We conducted a systematic review and qualitative meta-synthesis of the results, using grounded theory analysis methods (PROSPERO registration CRD42021262380), with literature published between January 2000 and February 2022. Seven databases were searched. Our review included literature about women with breech presentation, who sought a planned or unplanned VBB. Studies considering only experiences of alternative management (e.g. caesarean, external cephalic version), and those investigating healthcare workers’ experiences were excluded. Covidence systematic review software was used for screening and quality assessment. Qualitative data were extracted using NVivo software (20.5.0). Data were analysed through an iterative process based on constant comparison methods, with an iterative and reflexive code generation process. Codes were then arranged into ‘categories of experience’, which gave rise to over-arching themes.

**Results:**

Our review included 19 studies. We present one overarching theory: ‘Women who wish to plan a vaginal breech birth seek connected autonomy’. Our schematic, depicting this theory, includes seven main categories of experience: paternalistic healthcare; emotional turmoil; judgement and self-doubt; mother vs society: refusing to conform; isolated but united by breech; welcomed direction; and supported self-determination and self-efficacy.

**Conclusions:**

Women seeking to plan a VBB feel vulnerable and wish to connect with capable and confident healthcare providers. To meet their needs, services should be designed so that they can connect with clinicians who are willing and able to support their autonomy. Services should also seek to limit their exposure to disrespectful and judgemental interactions with healthcare providers.

## Plain English summary

Members of our public involvement and engagement group and some participants in the OptiBreech research reported difficulty in attempts to access supportive care when trying to plan a vaginal breech birth. This conflicts with national guidance, which emphasises choice and informed decision-making. We wanted to understand more about the experience of seeking to plan a vaginal breech birth, so we searched for research on this topic.

We made a careful plan before we started and registered this plan. We searched seven online databases for literature published between January 2000 and February 2022. We focused on studies about women pregnant with breech babies, who sought to plan a vaginal breech birth or whose baby was discovered to be breech in labour. We used Covidence systematic review software to organise and assess the quality of the research we collected. We gathered main themes and illustrative quotes from all of the papers and compared these. We met frequently to discuss our observations and to agree on how we would summarise information we gathered.

We agreed that, overall, women who wanted to plan a vaginal breech birth felt vulnerable. They wanted to connect to confident, capable healthcare providers who could help them achieve a vaginal birth as safely as possible: ‘connected autonomy.’ But their actual experiences ranged widely, including: paternalistic healthcare; emotional turmoil; judgement and self-doubt; mother vs society: refusing to conform; isolated but united by breech; welcomed direction; and supported self-determination and self-efficacy.

We concluded that, to meet the needs of women who wish to plan a vaginal breech birth, services should be designed so that they can connect with clinicians who are willing and able to support their autonomy. Services should also limit their exposure to disrespectful and judgemental interactions with healthcare providers.

## Introduction

Approximately 1:25 women (4%) experience pregnancy with a breech-presenting foetus at term ^1^. These babies are at higher risk of a poor outcome, regardless of mode of delivery ^1^. Most breech presentations are identified prior to labour, when United Kingdom (UK) and international guidelines indicate women should be offered management options to reduce this risk. These include external cephalic version (ECV) to turn the baby head-down ^2^, and discussion of choices about mode of childbirth, including a caesarean birth or vaginal breech birth (VBB) with experienced professionals ^1^. However, 20–30% of breech presentations remain undiagnosed until the onset of labour, in settings where universal presentation screening by ultrasound is not offered ^3^. The option of turning the baby is usually not available at this point, but guidelines emphasise choice and informed decision-making about mode of birth ^1, 4^.

The current Royal College of Obstetricians and Gynaecologists (RCOG) guideline suggests that with skilled and experienced support, a planned VBB can be nearly as safe as cephalic birth, but throughout the UK, this level of skill and experience is frequently either unavailable or inaccessible ^5, 6^. Available research about women’s theoretical and actual choices around mode of birth for a breech-presenting baby at term indicate as many as 35-58% of women may choose to plan a VBB when given the option, but this choice is highly dependent on the professional providing the counselling ^5, 7, 8^. This creates disparities between what women may wish to choose and what is available for them to choose. Such inconsistencies between expectations of maternity services and its realities are likely to negatively affect women’s experiences of pregnancy and childbirth.

The aim of this study was to explore the experiences of women seeking to plan a VBB, as described in primary research. We also aimed to synthesise this information into a theoretical model that could facilitate better understanding of this experience and how it might be improved.

## Methods

We conducted a systematic review and qualitative meta-synthesis of the results, using grounded theory analysis methods ^9, 10^. With this method, we sought to generate a model that could: 1) describe women’s experiences of planning a vaginal breech birth, and 2) offer strategies for improving this experience that were grounded in evidence.

Covidence systematic review software was used for screening and quality assessment. Qualitative data were extracted using NVivo software (20.5.0). Data were analysed through an iterative process based on constant comparison methods, with an iterative and reflexive code generation process. Codes were then arranged into ‘categories of experience’, which gave rise to over-arching themes.

The protocol was developed and registered on PROSPERO (reg. no. CRD42021262380). The review included literature about women pregnant with a breech-presenting fetus, who sought a planned or unplanned VBB, published between January 2000 and February 2022. Studies considering only experiences of alternative management, such as caesarean birth or external cephalic version, and those investigating healthcare workers’ experiences were excluded. Table 1 summarises the review’s Eligibility Criteria.

**Table 1.** Eligibility criteria. *Only studies that satisfied every element of the inclusion criteria were included. Studies were excluded if they fulfilled any 1 or more of the exclusion criteria*.

	Inclusion Criteria	Exclusion Criteria
**Population**	Mothers, parents, women and birthing people with breech presentation (either during pregnancy or at childbirth)	Any population other than our inclusion population (e.g. any healthcare professionals)
**Exposure**	Seeking a planned or unplanned vaginal breech birth	Study concerns ONLY experiences of external cephalic version, caesarean section, imaging or alternative management routes or interventions, without data concerning women’s experiences of breech pregnancy and childbirth.
**Outcome**	Our populations’ experiences of decision- making and support	Experience, attitudes, confidence or perspectives of any population other than our inclusion population
		Studies concerning outcome and impact of breech clinics, breech education or training of HCP's, or additional specialist services
		Study concerns trends, prevalence, indications, risk factors, outcomes, complications, morbidity, mortality, guidelines, protocols or management without data concerning women’s experiences of breech pregnancy and childbirth.
		Study concerns the cost-effectiveness of interventions
		Study concerns physiology
**Types of** ** studies**	qualitative, quantitative or mixed- methods research	RCT's, case-series, discussions, letters
**Context**	Study published in or after the year 2000, about human subjects, in English	Studies published prior to the year 2000

Seven databases were searched. The initial search was completed in July 2021; a follow-up search in February 2022 returned 1 new paper. A full Search Strategy is available in Table 2. Subject experts were contacted to identify key literature not yet published, to reduce publication bias. These sources were screened similarly to the database results.

**Table 2.** Search Strategy. *Search terms were formulated by identifying key words covering the criteria of PEO: Population (women), Exposure (vaginal breech birth) and Outcome (experience). Synonyms and variations of key words were then generated. Search terms were refined by running scoping searches*.

Key words - PEO		
Population: Women	Exposure: Vaginal breech birth	Outcome: Experience
Synonyms and variations		
Women*	Vaginal birth	Experience*
Mother*	VBB	Plan*
Matern*	Labour*	Cho*
Parent*	Deliver*	Elect*
	Pregnan*	Deci*
	Birth*	Prefer*
	Present*	Discuss*
	At term	View*
	Born	Attitude*
		Wish*
		Seek*

**Table:** Seven databases were searched:

Database Name	Dated database content	Date searched
Embase (Ovid)	1974 - 2021 week 23	June 2021
MEDLINE(R) AND Epub Ahead of Print, In-Process, In-Data-Review & Other Non-Indexed Citations and Daily (Ovid)	1946 to June 16, 2021	June 2021
Maternity & Infant Care Database (MIDIRS)	1971 to May 11, 2021	June 2021
Cochrane Library	All available dates - 2021 week 23	June 2021
AMED (Allied and Complementary Medicine)	1985 to June 2021	June 2021
PsychInfo	1806 to June Week 2 2021	June 2021
CINAHL	All available dates - 2021	June 2021

**Table:** **Ovid search strategy** (includes Embase, MEDLINE, MIDIRS, AMED and PsychInfo):

1	Mother* or women* or matern* or parent*.ab.
2	Vaginal birth or vaginal breech birth or VBB or labour* or deliver* or pregnan* or birth or present* or at term or born.ab.
3	Experience* or plan* or cho* or elect* or deci* or prefer* or discuss* or view* or expect* or attitude* or wish* or seek*.ab.
4	1 and 2 and 3
5	Breech.ti
6	Breech.ab
7	5 OR 6
8	4 and 7
9	Remove duplicates: 1530
Limits:	English language, human and humans.
Filters:	Year 2000-2021

**Table:** **Cochrane Library search strategy**:

1	Mother* OR women* OR matern* OR parent*
2	Breech
3	Vaginal birth OR vaginal breech birth OR VBB or labour* or deliver* or pregnan* or birth or present* or at term or born
4	Experience* OR plan* OR cho* OR elect* OR deci* OR prefer* OR discuss* OR view* OR expect* OR attitude* OR wish* OR seek*
5	1 and 2 and 3 and 4
Limits	None

**Table:** Each search included keyword, title and abstract.

1	Abstract: Mother* OR women* OR matern* OR parent*
2	Abstract: Vaginal birth OR vaginal breech birth OR VBB or labour* or deliver* or pregnan* or birth or present* or at term or born
3	Abstract: Experience* OR plan* OR cho* OR elect* OR deci* OR prefer* OR discuss* OR view* OR expect* OR attitude* OR wish* OR seek*
4	1 and 2 and 3
5	Title: breech
6	Abstract: breech
7	5 or 6
8	4 and 7
Limits	Human, english language, studies between 2000 - 2021

**Table:** **CINAHL search strategy**:

1	Abstract: Mother* OR women* OR matern* OR parent*
2	Abstract: Vaginal birth OR vaginal breech birth OR VBB or labour* or deliver* or pregnan* or birth or present* or at term or born
3	Abstract: Experience* OR plan* OR cho* OR elect* OR deci* OR prefer* OR discuss* OR view* OR expect* OR attitude* OR wish* OR seek*
4	1 and 2 and 3
5	Title: breech
6	Abstract: breech
7	5 or 6
8	4 and 7
9	Limits: Human, english language, studies between 2000 - 2021

**Figure 1.**
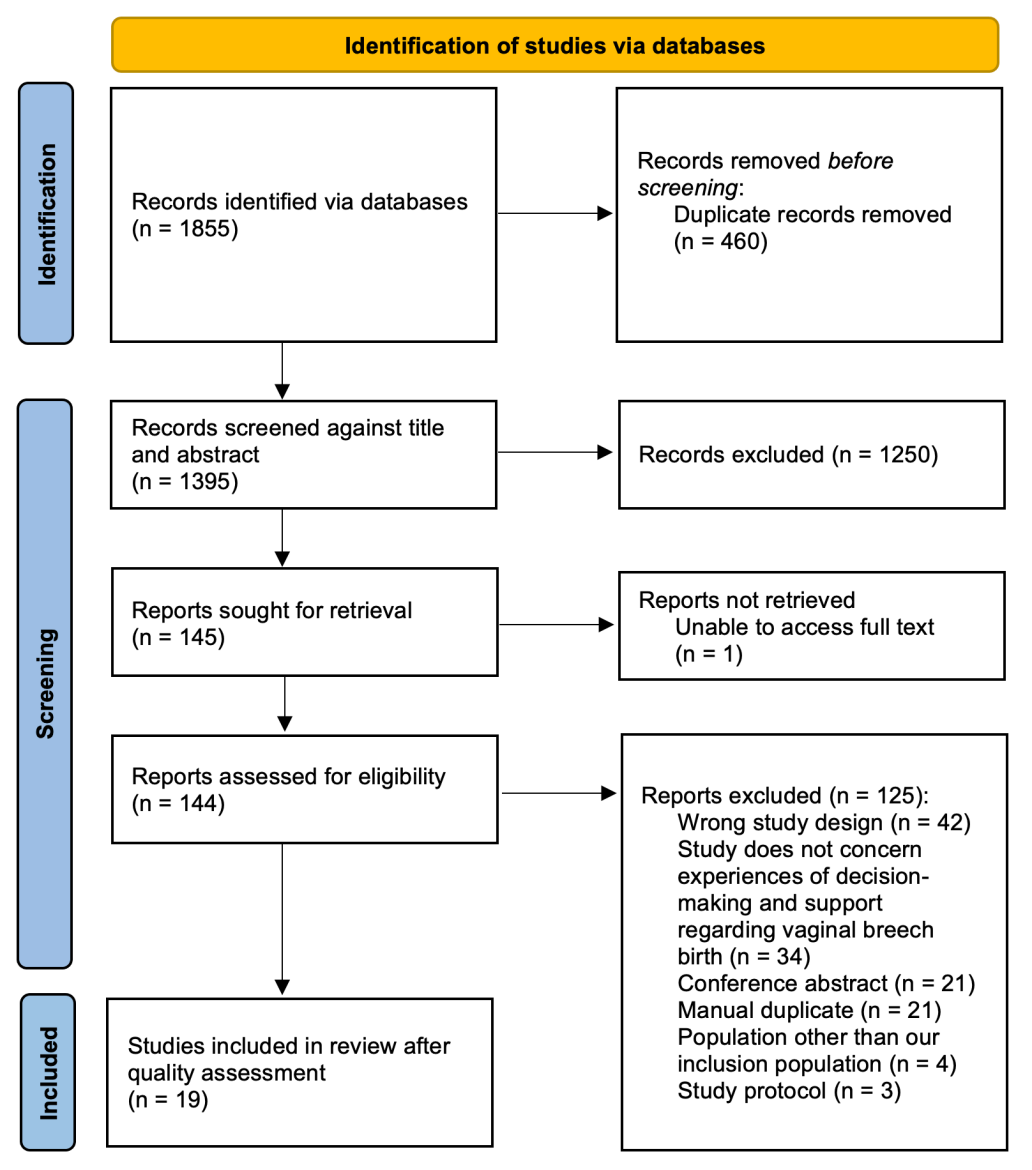
PRISMA flow diagram.

Covidence systematic review software was used for screening and quality assessment. Two reviewers (RR and CG) carried out eligibility screening and quality assessment of the data independently, consulting a third reviewer (SW) to resolve conflicts. The results of this process are reported in Figure 1: PRISMA Flow Diagram ^11^. Studies were scored to assess bias, using a points system adapted from the CASP Tool (2018) checklist, with 1 point per criteria fulfilled. Studies scoring 5 or less after a consensus was reached were excluded. Data extraction was also performed independently in Covidence, using a generated template, available in Table 3.

**Table 3.** Covidence data extraction template.

General information	
Study ID	
Title	
Lead author contact details	
Country in which the study conducted	☐ United States ☐ UK ☐ Canada ☐ Australia ☐ Other
Notes	
Characteristics of included studies	
Methods	
Aim of study	
Study design	☐ Randomised controlled trial ☐ Non-randomised experimental study ☐ Cohort study ☐ Cross sectional study ☐ Case control study ☐ Systematic review ☐ Qualitative research ☐ Prevalence study ☐ Case series ☐ Case report ☐ Diagnostic test accuracy study ☐ Clinical prediction rule ☐ Economic evaluation ☐ Text and opinion ☐ Other
Start date	
End date	
Study funding sources	
Possible conflicts of interest for study authors	
Participants	
Population description	
Inclusion criteria	
Exclusion criteria	
Method of recruitment of participants	☐ Phone ☐ Mail ☐ Clinic patients ☐ Voluntary ☐ Other
Total number of participants	

Qualitative data was extracted using NVIVO software (20.5.0). Data were coded independently by RR and CG, with regular meetings to discuss emerging codes. Codes were generated by constant comparison of the data and developing codes, in a reflective and iterative process, with the reviewers re-coding as necessary throughout. The data were then re-coded using the finalised codes.

**Figure 2.**
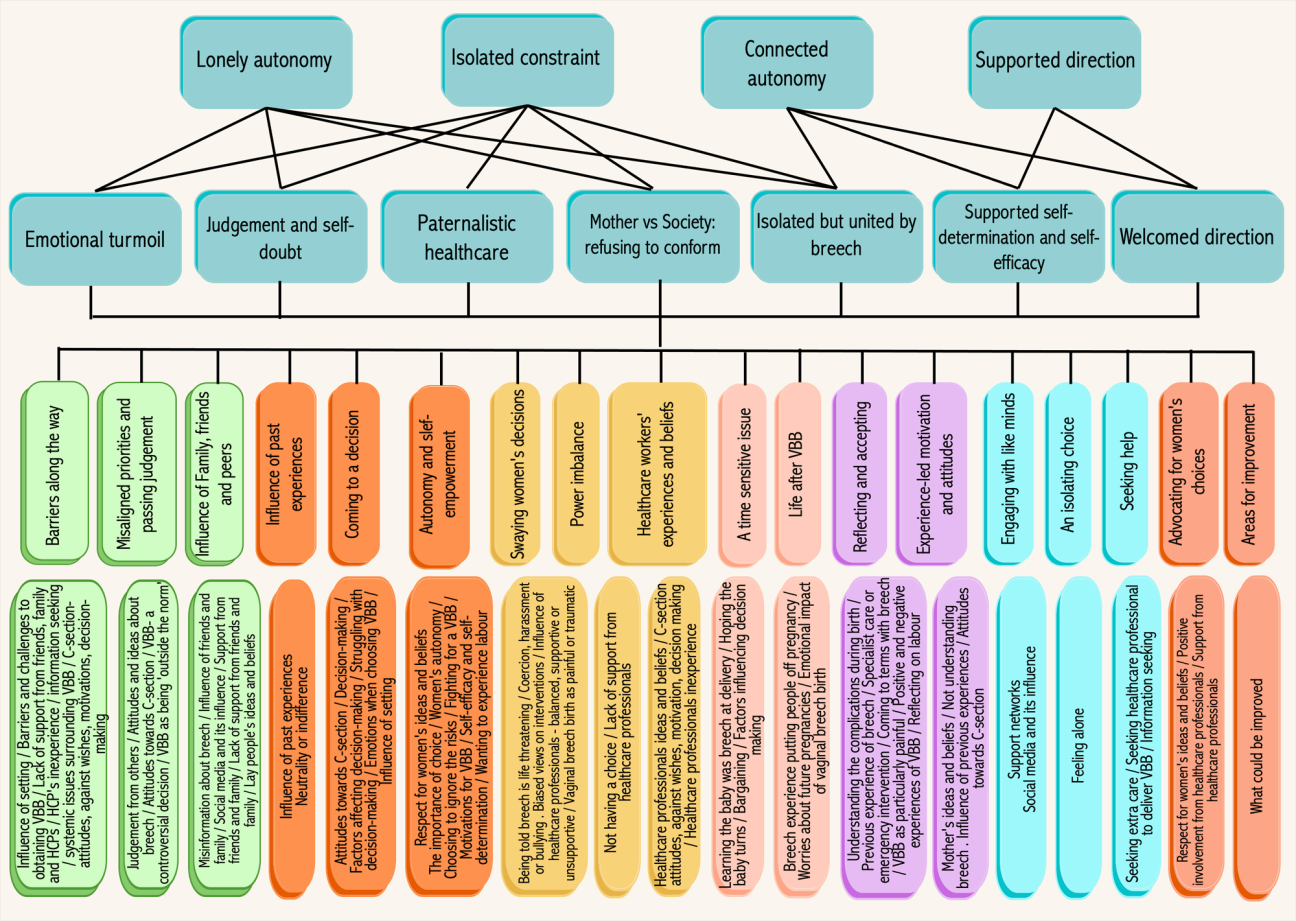
Concept map tree diagram.

Codes were organised into ‘categories of experience’ and relevant sub-categories, reflecting themes within those categories. This involved discussion between the review team and constant return to the data. This process is shown in Figure 2: Concept Map. The lay member of the team, who also had access to the primary sources, checked the resonance of our emerging findings and highlighted key areas needing further analysis. A grounded theory was developed to explain the way in which the identified categories and themes interacted.

## Results

Our search returned 1395 studies once duplicates were removed. One study was discussed by all 3 reviewers after scoring 5 at quality assessment; the decision was made to include the study due to its value in exploring breech birth decision-making. Studies included qualitative (14), mixed (4) and systematic review (1) methods. Nine countries and international on-line samples were represented. Lead authors included midwives (8), service users (6) and obstetricians (5). Characteristics of included studies are summarised in Table 4. 

**Table 4.** Characteristics of Included Studies

Authors Date Journal	Title	Aim of study	Study design Role of lead author	Population Setting	Inclusion criteria	Exclusion criteria	Recruitment method	Number of participants	Quality Assessment Score
Davidson 2015 PhD thesis	The experience of vaginal breech birth: A social, cultural and gendered context	To explore the complex meanings of the experience of breech, and to understand this experience within a social, cultural and gendered context.	Qualitative Midwife	Women who received care from a National Health Service hospital or an independent midwife, who have had a vaginal breech birth in the previous 5 years. Health care professionals. United Kingdom	Childbearing woman who had given birth vaginally to a live singleton breech baby in the last 5 years. Currently registered health professionals who had provided care for women during a VBB in the last 5 years. Speaks and understands English.	Women that have been provided care by the researcher. Health professionals who had been taught by the researcher in the previous 5 years. Health professionals undergoing any form of supervisory review or investigation .	Letter and phone for mothers. Health professionals recruited on voluntary basis.	11 women, 6 midwives and 2 obstetricians.	10
Founds 2007 *Int J of Nursing* * Studies*	Women's and providers’ experiences of breech presentation in Jamaica: A qualitative study	To increase the understanding of womens and providers experiences of breech presentation and to understand the effects of context on these experiences.	Qualitative Midwife	Women under the care of one rural hospital and staff working at the hospital. Jamaica	Postpartum women who birthed singleton live born breech infants in the past year. Experienced obstetric care providers.	No data	Referrals and independent contact led to interviews with women and clinicians	14	10
Glasø, Sandstad and Vanky 2013 *Acta Obstet * *Gynecol * *Scand*	Breech delivery- -what influences on the mother's choice?	To investigate factors influencing mother's choice of delivery mode when vaginal breech delivery is considered possible and safe.	Retrospective mixed methods Obstetrician	Women who had a breech presentation, external version of breech presentation or trial of external version of breech presentation. Norway	Live, term singleton fetus (gestational age 37+0) in breech presentation delivered at the hospital during the study period, in whom vaginal delivery was considered possible and safe.	Exclusion criteria were multiple pregnancies, gestational age<37+0 and suspicion of foetal anomaly or malformations.	Mail	204	9
Guittier *et al*. 2011 *Midwifery*	Breech presentation and choice of mode of childbirth: a qualitative study of women's experiences.	To explore women's perceptions of their experience of the diagnosis of breech presentation and decision-making processes regarding the choice of mode of childbirth.	Qualitative Midwife	Pregnant women diagnosed with a singleton fetus in the breech position under the care of the maternity unit of the University Hospitals of Geneva. Switzerland	Persistent breech presentation after the 38th week of pregnancy, fluency in the French language, and no medical or obstetric contraindications for vaginal childbirth.	No data	Phone	12	9
Homer *et al*. 2015 *BMC* * Pregnancy * *and * *Childbirth*	Women’s experiences of planning a vaginal breech birth in Australia	To explore the experiences of women who had planned a vaginal breech birth in Australia in the preceding seven years	Qualitative Midwife	Women from two hospitals which were public maternity units in urban/metropolitan areas that planned a vaginal breech birth. Australia	Women who planned a VBB for a singleton pregnancy in the past seven years regardless of the eventual model of birth and could read and speak English	No data	Women attending clinic	22	10
Kok *et al*. 2008 *Patient * *Education* * and* * Counseling*		To assess expectant parents preferences for mode of delivery in case of term breech position, and their judgment of the neonatal short- and long-term risks as well as the maternal risks	Mixed methods Obstetrician	Women who had an otherwise uncomplicated singleton pregnancy 36 weeks and onwards, and their partners. Netherlands	Women who had a breech or cephalic pregnancy from 36 weeks onwards, and their partners if present.		Women attending clinic, including 40 women and 6 fathers with breech pregnancy and 40 women and 21 fathers with cephalic pregnancy	107	9
Lightfoot 2018 PhD thesis	Women’s experiences of undiagnosed breech birth and the effects on future childbirth decisions and expectations	To give voice to women who have experienced an undiagnosed breech birth and to consider the influence this experience may have had on decisions about future pregnancy and childbirth and the associated expectations women may have.	Qualitative Service User	Mumsnet discussion boards relating to undiagnosed breech pregnancy. International	Thread started any time after and including 1st September 2012. Thread to explicitly mention undiagnosed breech birth experience belonging to the individual posting. Any comments from a poster identified as having an undiagnosed breech birth from one post.	All messages in a thread containing no explicit information on an experience of undiagnosed breech birth. Second-hand stories of breech birth.	Social media threads obtained from advanced Google Search Function	45 women, 44 relevant threads, 1364 messages.	10
Molkenboer *et al*., 2008 *J Psychosom* * Obstet * *Gynaecol*	Mothers' views of their childbirth experience two years after term breech delivery	To evaluate mothers’ views of their childbirth experience two years after term breech delivery	Mixed methods Obstetrician	Women with a term breech presentation between July 1998 and April 2000. Netherlands	Women with a term breech presentation between July 1998 and April 2000 who did not participate in the TBT and were not randomized.	Women who participated in the TBT and women who had children born with lethal congenital anomalies.	Women attending clinic	183	7
Morris, Geraghty, and Sundin 2021	Women’s experiences of breech birth and disciplinary power	To explore women's experiences of breech pregnancy and birth to identify areas in practice for improvement.	Qualitative Midwife	Women who had had a breech birth in Western Australia. Australia	At least 18 years of age, English speaking and had experienced a live breech birth in WA at 36 or more weeks gestation.	<36weeks gestation, successful External Cephalic Version followed by a cephalic birth or did not give birth in Western Australia.	Voluntary recruitment via social media	20	10
Morris, Sundin and Geraghty 2022 *European J* * of Midwifery*	Women’s experiences of breech birth decision making: An integrated review	To integrate current knowledge surrounding women’s experiences of breech birth decision- making, obtained from a systematic search of the literature, in order to highlight potential practice improvements	Systematic review Midwife	Published literature relating to the topic. Population was women with a breech presentation at term. International	Written in english, full text, peer-reviewed articles published between 2012 and 2021.	Not meeting the selection criteria, focussing only on experiences or outcomes of an intervention such as External Cephalic Version (ECV) or CS	Database search terms	8 studies were included in qualitative synthesis.	9
Petrovska, Sheehan and Homer 2017 *Women and* * Birth*	The fact and the fiction: A prospective study of internet forum discussions on vaginal breech birth	To examine how women use English language internet discussion forums to find out information about vaginal breech birth and to increase understanding of how vaginal breech birth is perceived among women.	Qualitative Service User	Women using English language internet discussion forums to discuss VBB International	Google alert for the terms 'breech birth' and 'breech'. Posts in the period 1/1/13 - 31/12/13.	No data	Google alert for the terms 'breech birth' and 'breech'.	50 forum discussions containing 382 comments.	10
Petrovska, Sheehan and Homer 2017 *J of Midwifery* * and* * Women's* * Health*	Media Representations of Breech Birth: A Prospective Analysis of Web- Based News Reports	To explore the content and tone of news media reports relating to breech presentation and breech birth	Qualitative Service User	News reports on the internet sourced from google alerts to include the terms breech and breech birth. International	Sampling was limited to reports from media outlets reporting on all aspects relating to breech presentation and breech birth.	Internet chat forums and personal blogs were excluded	Google alerts for the search terms 'breech' and 'breech birth	138 web- based news reports	10
Petrovska *et al*. 2016 *Birth*	Supporting Women Planning a Vaginal Breech Birth: An International Survey	To explore the experiences of women who reported choosing a vaginal breech birth and were motivated to seek supportive care and information that assisted them to access this option for birth. This study also aimed to increase understanding in how to best support these women and provide quality information.	Qualitative Service User	Women who reported choosing a vaginal breech birth, who were a part of closed membership Facebook groups from the US, UK and Australia that had a focus on VBB. Women who were interviewed for another study were invited to participate. International	Women who had previously planned a vaginal breech birth.	No data	Online survey on closed membership Facebook groups and women who were interviewed for the original research conducted on decision making experiences for VBB.	204	9
Petrovska *et al*. 2017 Midwifery	‘Stress, anger, fear and injustice’: An international qualitative survey of women's experiences planning a vaginal breech birth	To examine the views and experiences of women from a number of high-income countries who sought a VBB, with a view to increase understanding as to how these women can be best supported should they choose this option for care	Qualitative Service User	Women who have planned a vaginal breech birth at or close to term in the past 7 years. International	Members of closed membership Facebook groups from the United States, United Kingdom and Australia that had a focus on VBB and whose membership to these groups is not limited to women from these countries. Women who were involved in previous research on women's experiences in planning VBB undertaken by the authors.	No data	Social media, via closed Facebook groups.	204	9
Petrovska *et al*. 2017 *Health, Risk* * and Society*	How do social discourses of risk impact on women’s choices for vaginal breech birth? A qualitative study of women’s experiences	To explore the impact of social discourses of risk around childbirth on the decisions made for birth by women who planned to have a breech baby late in pregnancy.	Qualitative Service User	Women who planned a vaginal breech birth at a large metropolitan hospital in N.S.W, Australia who were cared for by a clinician. Australia	Women who planned a vaginal breech birth for a singleton pregnancy in the previous 7 years regardless of their eventual model of birth. More than 37 completed weeks gestation at the end of their pregnancy. Could read and speak English. Were available for a face-to-face interview after the birth.	No data	Women attending clinic	22	10
Thompson, Brett and Burns 2019	What if something goes wrong? A grounded theory study of parents’ decision-making processes around mode of breech birth at term gestation	To explore factors that influence parents term breech mode of birth decision-making within the NHS care model.	Qualitative Midwife	Women who were presenting or had presented with breech birth at term. Pregnant women, post-natal women and their partners. United Kingdom	Parents self-reporting a singleton breech baby confirmed by ultrasound at 36+0 weeks gestation, who were at least 16 years old and spoke sufficient English to consent to and participate in interviews.	No data	UK social media, including Facebook, MumsNet, and Mums Advice	12	10
Toivonen *et al*., 2014 *Birth*	Maternal Experiences of Vaginal Breech Delivery	To compare birth experiences between breech and vertex deliveries and to identify the risk factors for an unsatisfactory birth experience.	Mixed methods Obstetrician	Mothers intending vaginal breech deliveries between January 2008 and October 2012 at Tampere University Hospital. Vertex controls also selected. Finland	Breech delivery between Jan 08 and Oct 2012. Each next delivery recorded in the delivery room records after the intended breech delivery.	Mothers who have given birth since their breech birth.	Mail	170	9
Wang, Cotter and Fahey 2021 *J of Obst and* * Gyn Canada*	Women's Selection of Mode of Birth for their Breech Presentation	To clarify the decision- making process and the supports and barriers that women face when diagnosed with a breech presentation in a region that has options available for mode of birth.	Qualitative Obstetrician	Women who gave birth to a breech-presenting baby Canada	Women who had birthed a breech fetus from 4 hospitals in Calgary, Alberta between Jan 1st and April 30th 2016.	No data	Women attending clinic	95	5
Watts *et al*., 2016	This baby is not for turning: Women’s experiences of attempted external cephalic version	To examine women's experience of an ECV which resulted in a persistent breech presentation.	Qualitative Midwife	Women who had an unsuccessful ECV, from two Australian public maternity units in urban/metropolitan areas that supported women to have a vaginal breech birth. Australia	English-speaking women, who after an unsuccessful ECV planned a vaginal breech birth for a singleton pregnancy in the past 7 years regardless of their eventual mode of birth	No data	Women attending clinic	22	8

**Figure 3.**
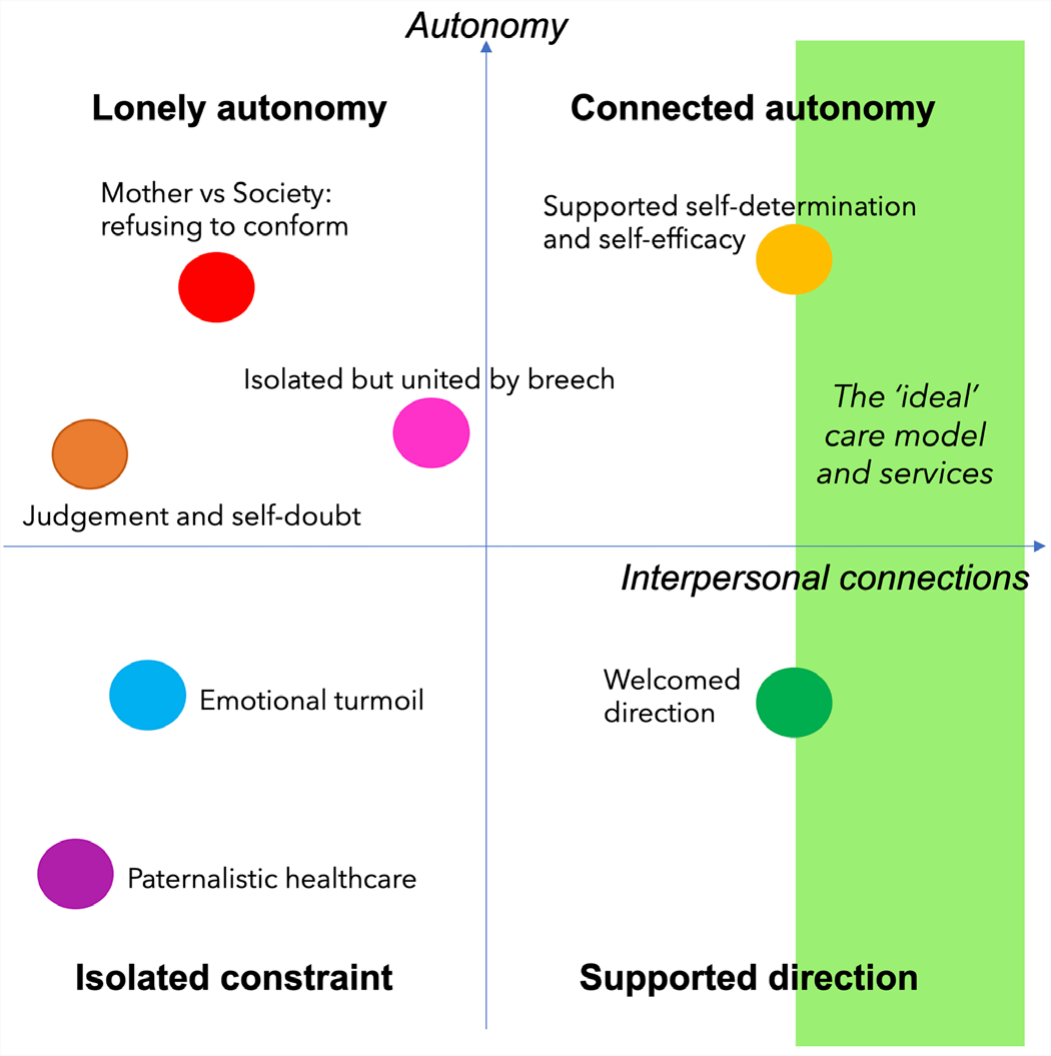
Connected autonomy schematic.

We approached our meta-synthesis using the constant comparative methods of grounded theory ^10, 12^. Our initial analysis generated 94 codes, from which we developed categories and subcategories classified by types of experiences. These included seven main categories: paternalistic healthcare; emotional turmoil; judgement and self-doubt; mother vs society: refusing to conform; isolated but united by breech; welcomed direction; and supported self-determination and self-efficacy. Considering the sum of the data, we developed an overarching theory: *Women who wish to plan a vaginal breech birth seek connected autonomy*. We organised the categorical experiences of seeking to plan a VBB on a schematic axis, reflecting how women’s feelings of connection and support intersected with their ability to exercise personal autonomy. This is represented in Figure 3: Connected Autonomy Schematic.

### Paternalistic healthcare

In the ‘isolated constraint’ quadrant of our schematic, women’s experiences were characterised by disconnection from their healthcare providers and little ability to make autonomous decisions about mode of birth.

In many instances, women were denied the choice of a VBB: “There was a void between the information they received and the information they wanted. For the most part, an alternative to CS was not provided.” ^13(p4)^ Where the option was mentioned, it was accompanied by highly emotive and frightening descriptions of breech births that had gone wrong: “You don’t have a choice, your babies are going to die, you are going to die, why did you come here if you don’t want us to help you, your kids will be left without a mother …” ^14(p43)^

It was a priority for most women that they “retained control of decision-making, retained a sense of personal choice and retained the option of having a vaginal birth,” ^15(p10)^ but this did not happen in many cases. Many women felt their rights and choices were forcibly denied: “It was the most disempowering experience of my life.” ^14(p43)^ “We discover that our experience isn’t worth anything and we should just be listening to what we are told.” ^16(pe212)^

This created disconnect between women seeking a VBB and their providers. Some women perceived the support from encouraging healthcare professionals to be curtailed by their colleagues, which undermined their trust in the healthcare team. Women expected providers to have adequate training to support VBB but found this was often not the case: “I feel it's a shame there is not more education and support for new doctors coming through. They can't support us mums of breechlings if they aren't supported themselves.” ^14(p45)^ This highlights the imbalanced power dynamic between mothers and healthcare professionals, and amongst professionals themselves, that enforces paternalistic care.

Even in settings where VBBs were more common, some of this lack of control persisted. Women planning a vaginal birth reported lack of involvement in decision-making more often (29.6% versus 2.9%) ^17^ and fewer felt the final decision on mode of delivery was theirs rather than the doctor’s (60% versus 85%) ^18^, compared to women planning a caesarean birth. Toivanen et al reported the only significant difference between the experience of women attempting breech and vertex births was a significantly reduced sense of participation in their birth ^19^.

### Emotional turmoil

Experiences of ‘isolated constraint’ also led to significant ‘emotional turmoil.’ Women felt strong negative emotions when receiving their breech diagnosis, including fear and anxiety ^14, 16, 18– 22^, uncertainty ^16, 23– 25^, powerlessness ^23, 25^ and grief: “I don’t talk about it I guess but when I do I get quite upset. It has probably upset me more than I realise... it took me quite a while to get over it.” ^25(p131)^ Their emotional turmoil was exacerbated when women were not given an opportunity to express their feelings and wishes ^23^.

Women’s emotions surrounding breech were influenced by their own experiences, and those of people around them. Family pregnancy and childbirth experiences were especially important, with unresolved trauma from previous experiences generally influencing mothers to opt for planned caesarean birth.

Women also felt that breech diagnosis was ‘a time-sensitive issue’. Transitioning from passive to autonomous decision-making required information, which took time to receive or seek out. The timing of breech diagnosis often played a role in mothers’ reactions and decisions. Discovering breech presentation during labour was often “chaotic and traumatic.” ^26(p3127)^ Many women felt they missed the chance to be better prepared, and most studies found that women preferred earlier diagnosis of breech presentation ^24^.

### Judgement and self-doubt

Most women who actively tried to plan a VBB experienced ‘judgement and self-doubt,’ an experience of ‘lonely autonomy.’ Women reported frustration that they were considered “not capable of making the right decision” ^15(p9)^ about their birth and feeling devalued as part of their own healthcare. Enhanced communication was an important and recurrent suggestion for improvement, as women often felt their motivations for a VBB were misunderstood.

In addition to healthcare professionals, “pressure and judgement from family members” ^14(p43)^ also influenced the decision-making process ^16, 20– 22, 24– 28^. Many felt “upset when family and friends accused them of being selfish and ‘putting the birth before the baby.’” ^15(p8)^ “People in women’s social networks related to the women that breech presentation posed life-threatening risks for mother and baby.” ^22(p1394)^ Avoiding the self-doubt this engendered required women to distance themselves from others: “I switched off to it because I was confident with my decision.” ^15(p7)^ One woman shared how she withheld information about her birth plans from friends and how she “disengaged from the ‘ones that doubted.’” ^25(p141)^

Women felt alone in assuming responsibility for their decision: “It’s a decision where you are fundamentally alone at the end. Even if there are people who are there with you, you’re finally the only one to have to assume such a decision. Because... , finally, we are the only person who must push and must expel the infant.” ^16(pe212)^ Where they ultimately chose to plan a caesarean birth, often women still felt misunderstood and alone with their grief. One woman explained “And the fact that I thought I was going to miss out on that part, I was already grieving it. So the grief - nobody really understood the grief apart from my partner.” ^13(p4)^

### Mother vs society: refusing to conform

This category was also characterised by ‘lonely autonomy’. Women with plans for a VBB encountered resistance and barriers at all levels: social circles, clinicians, and institutional policies: “To try for a natural breech birth and go against the status quo - that’s a really hard thing to do.” ^15(p10)^ Many possessed strong self-determination and self-efficacy at odds with systems that failed to accommodate this. Many women felt they were ‘fighting’ the people and systems who were supposed to be caring for them.

In “‘going against the tide’ of current practice,” ^15(p23)^ most women also had to “circumvent a system that was blocking them from attempting a VBB.” ^13(p5)^ Where local providers were inexperienced or unwilling, choosing not to have an elective caesarean birth meant women had to transfer hospitals to access a VBB. Women “seeking a VBB highlight[ed] the lack of supportive/adequately experienced clinicians as a barrier to achieving the birth that they wanted.” ^26(p3129)^ Some women were forced to travel long distances ^13– 15, 22, 24– 26, 28^, introducing a physical disconnect between them and their primary providers.

After overcoming these challenges, women often encountered additional institutional barriers, such as policies preventing experienced providers from caring for them, at all or outside of regularly scheduled working hours. Although balanced information made a significant difference to women’s decision-making ^7, 29^, many women encountered systemic barriers to accessing balanced, evidence-based information ^21^.

### Isolated but united by breech

Some women found support in social media networks. These served as an “alternative support system” ^15(p13)^ to women experiencing judgement and isolation from their usual social networks after deciding to pursue VBB. Women accessed social media during their information seeking activities, finding it helpful to “read birth stories and get good support online from breech moms across the globe” ^14(p44)^ which in turn, often helped to alleviate anxiety ^23^. For many, participation in these networks was influential in their decision-making process, with the effects of social media ranging from “an additional support” ^20(p119)^ to “hugely helpful and motivating when making [their] decision” ^14(p44)^. They used these forums to counter the isolation they were experiencing and seek validation and assurance.

### Supported self-determination and self-efficacy

A small minority of women had experiences of planning a VBB that were characterised by ‘supported self-determination and self-efficacy,’ an experience of ‘connected autonomy’. These women received care that aligned with their expectations of how services should operate: “My appointment with the specialist went so well today! … It’s all systems go for an active breech birth! Woohoo! He didn’t even question my decision. He just spoke to me like it was a done deal and made me feel so confident … I’m so relieved!” ^27(pe99)^

Multiple papers described how women sought out clinicians, described as ‘specialist[s]’ ^14, 20, 24– 27, 30^, who presented them with unbiased information and encouraged them to be an active part of decision-making, even if this meant changing providers. Finding this person significantly alleviated many women’s anxieties and boosted their personal feelings of self-efficacy in decision-making and birth. Women also highlighted continuity of carer as an important factor in building a trusting relationship. Midwives were often seen as ‘allies’, with the ability to navigate the healthcare system to meet mothers’ needs.

Women who attempted a VBB experienced a range of emotions, very similar to their counterparts who experienced cephalic birth ^19^. Some who achieved a planned VBB felt proud of their birth experience and recalled the importance of being ‘fully-engaged’ ^25(p141)^ in the birthing process. In many cases, the eventual outcome of the birth route was less important than having the choice and opportunity to try for a VBB, of ‘having a go’ ^13^.

Multiple studies concluded that “experience of healthcare professionals is also a key characteristic that is perceived to increase the likelihood of a more positive experience and a more favourable outcome to [a] breech birth” ^23(p77)^. Some doctors were honest with women about their personal inexperience, and supported them to find a clinician who could provide them the care they wanted: “Once I said to her I wanted to change to someone who would give me a chance she said ‘(names another private OB) he'll let you give it a go and so it's not that I believe that you can't do it but I’ve never done one myself.” ^26(p3126)^

### Welcomed direction

While most women sought active decision-making roles about their care, some preferred to defer to their care provider, welcoming their direction. This experience fell in the ‘supported direction’ quadrant of our schematic.

Some women preferred not to share decision-making; they preferred clear advice from professionals: “It was their decision not mine which was fine. It [would have been] much more difficult had I had to make the decision.” ^25(p151)^ This preference appeared to apply to both caesarean and vaginal birth: “…I’ll be scheduled for a c-section on Wednesday. Personally I wouldn’t deliver him vaginally … my hospital don’t even consider vaginal breech delivery! So I couldn’t have one if I wanted to.” ^27(pe99)^

Although most women expressed a preference for earlier, antenatal diagnosis of breech presentation ^23^, a small minority preferred their late diagnosis. These were mostly women whose baby’s breech presentation was diagnosed during a labour that ended in a healthy vaginal birth. This is because they knew less of the potential complications, which they perceived would have negatively impacted their experience. For example, one mother, “claimed she would have been afraid during labour, if she knew that breech could be life threatening.” ^22(p1395)^

We placed this category in a lower quadrant due to the lack of autonomy exercised by women in decision-making. The women themselves chose to pass this responsibility to their care providers which is distinguished from the experience of ‘paternalistic healthcare,’ where women experienced their right to choose as withheld.

## Discussion

### Main findings

Our study highlights the need for women to experience ‘connected autonomy’ and ‘supported direction’ during breech pregnancy and childbirth. Most women prefer autonomy over their mode of delivery and want non-judgemental support from skilled healthcare professionals regardless of their choice of birth mode. Most women value discussing their priorities with their healthcare providers and receiving balanced advice, i.e., supported direction.

Unfortunately, our review found that many women experience lonely autonomy and isolated constraint. In fighting for their birth choices many women found themselves alone and unsupported. Complying with the advice of their healthcare professionals, when in direct conflict with their preferences, led to feelings of regret and dissatisfaction.

Despite guidelines recommending that women be offered a choice regarding their delivery, our research indicates that many women found the choice of VBB is neither offered nor available. Even when willing and/or skilled birth attendants are available, systems are not set up to facilitate this. As a result, many women feel disconnected from their healthcare providers when they are most in need. 

## Strengths

This study benefits from careful consideration of bias throughout the research process. All screening, bias assessment, and data extraction were performed by both reviewers independently before discussion to reach a consensus, to prevent selection and interpretation bias. The two primary reviewers were medical students, and the senior supervising member of the review team was a midwife. Inclusion of studies recruiting social media or blog posts prevented sampling bias and reduced the effect of primary researchers on participants. Experts were consulted to identify key literature not retrieved by the literature search (for example, grey literature) to reduce publication bias.

Throughout the data coding and theme developing process, a service user was consulted to check resonance of the emerging findings. This further reduced interpretation bias and increased the transferability of our findings. They also helped to ensure our results were presented with balance and accuracy, in a way likely to be acceptable to women in our population of interest.

## Limitations

The strict eligibility criteria of the study may have reduced the scope of our review, and the search of databases for literature may not have retrieved all relevant results. The findings of this study may be less applicable to healthcare contexts that differ from those included in the study, as the nature of women’s experiences of VBB is both deeply personal and highly dependent on the services accessible. Only English language studies were included.

## Interpretation

Our study included literature from a wide variety of settings, including some settings that have frequent VBB. In settings where VBB was less common, conflict with healthcare providers appeared more frequent. While our study focused on the experience of planning a VBB, evidence from the included studies indicated that women’s dissatisfaction arose from lack of support for their preferred mode of birth and ability to make autonomous, informed decisions. Multiple studies have reported high levels of satisfaction when women’s request for a planned caesarean section is fulfilled, despite higher levels of postpartum complications when there is no medical indication ^18, 31^. In the few studies completed in settings where VBBs were common, feelings of lack of control and involvement in decision-making were still more common when compared to women planning vertex or caesarean births. This may be because of the historical focus on ‘selection criteria’ for selecting women who are ‘good’ candidates for VBBs. Such criteria often fail to align with women’s own feelings about their birth choices, which should be at the core of person-centred healthcare.

We hope that this review will help healthcare providers to understand the experiences of women who seek to plan a VBB at term. Although this choice is often viewed in opposition to the ‘mainstream’, in which most women are advised to and choose to plan a caesarean birth for their term breech baby, a significant number of women only reluctantly choose this position. Women who wish to plan a VBB also want to work in partnership with supportive, skilled providers. Services can and should do more to ensure they are able to do this.

## Implications for future practice, policy and research

Our findings suggest that specialist services dedicated to breech births would be beneficial for mothers. There have already been previous calls for such services ^24, 32^, and currently some integrated care pathways are in development ^33, 34^. Streamlined pathways would increase access to expert advice, and clinicians should receive training on communication and promoting shared decision-making. Communication may be improved by incorporating an individualised approach encompassing women’s values. Up to date, balanced and evidence-based information resources should be made available as a part of improving services. Decision aids may prove useful to women who feel overwhelmed by new information ^35^.

Women also sought social support from other mothers with similar experiences. The formation of support groups and forums dedicated to breech mothers, supported with input from breech experts, may enable shared experiences and accurate information relating to the evidence base. It is critical to improve the knowledge of healthcare providers regarding VBB; clinicians should receive training both to counsel breech mothers (communication training) and to perform safe VBB (skills-based training) to widen access to VBB ^36^.

## Conclusions

Our review aimed to explore women’s experiences of planning a VBB. Our findings show that women seek connected autonomy and supported direction yet often encounter lonely autonomy and isolated constraint. While there is a demand for VBB, women frequently feel pressured to accept the more mainstream option of caesarean birth and struggle to access supportive services. We found that women felt misunderstood in their motivations behind seeking VBB and felt alienated from a society labelling them ‘selfish’. Women felt communication with clinicians was vital to their birth experience and turned to their peers for support and to seek shared experiences.

Ensuring women have 'connected autonomy’ is vital to improving the breech birth experience. Women are vulnerable during this time and seek capable and supportive clinicians to help them achieve the birth they desire. Our recommendations include dedicated breech specialist pathways, increased availability of balanced, reliable educational resources for mothers, and improvement of training for clinicians to ensure wider access to experts in breech presentation and VBB. Further research will be needed to explore the clinical and cost effectiveness of these potential solutions.

## Ethical approval

As this was a systematic review and did not concern any primary or non-anonymised data, no ethical approval was sought.

## Data Availability

All data underlying the results are available as part of the article and no additional source data are required. Figshare: Women's experiences of seeking to plan a vaginal breech birth -- PRISMA reporting guidelines checklist, https://doi.org/10.6084/m9.figshare.21333252.v1 ^37^. Data are available under the terms of the Creative Commons Zero "No rights reserved" data waiver (CC0 1.0 Public domain dedication).
