# Supplementary material for: What are women’s experiences of seeking to plan a vaginal breech birth? A systematic review and qualitative meta-synthesis
Source: NIHR Open Res. 2023 Jan 20;3:4. [Version 1] doi: 10.3310/nihropenres.13329.1 (PMC10593332; doi:10.3310/nihropenres.13329.1)
Supplement: Peer review history

# Reviewer report: Reviewer response for version 1

**DOI:** 10.3310/nihropenres.14455.r29966

**Published:** 2023-09-07

**Authors:** Megan Cooper

**Affiliations:** 1 Flinders University, Adelaide, South Australia, Australia

Thank you for the opportunity to review this manuscript which examines the experiences of women who seek a vaginal breech birth. This is a much needed examination that has significant implications for care across the globe, especially with respect to women's autonomy. 

 The paper is a pleasure to read and is extremely well written. This is a credit to the authors. Minor recommendations and suggestions are provided below.

 Within the plain English summary there is reference to the categories but they are not referred to as such. I would suggest that you refer to the findings as categories as you have throughout the paper to avoid any confusion. 

 In the first paragraph in the introduction, there is reference to women being offered management options to "reduce the risk." Do you mean the risk of breech presentation or the risks associated with experiencing a breech birth?

 Can you explain the rationale for the inclusion criteria from January 2000 and February 2022? Following the Term Breech Trial, much changed. Was this the reason? I would suggest that it would be worthwhile to provide the rationale. 

 "Constant return to the data" on page 6 reads a little clumsy. I am also wondering whether "interacted" is the right word when referring to relationship between categories and themes?

 While a very different focus, I can draw very strong similarities between these findings and the barriers and challenges that women experience in accessing water immersion for labour and birth and other options that are not mainstream or well-accepted. Is there scope for these similarities to be drawn to emphasise that the issue transcends vaginal breech birth and is reflective of the state of maternity services more broadly? That is, women's autonomy is only supported where their decisions align with what the individual practitioner, institution and/or policy support. 

 Page 9, second paragraph under "Emotional turmoil" - are you referring to unresolved trauma relating to family experience of breech birth or birth more generally?

 Given the generalised lack of support for vaginal breech birth, is it more important that there is greater education to overcome the negativity rather than services that are specific to breech? There is clearly an aversion and while the premise of specialised breech clinics/services may address the issue of women's access to a supportive clinician, it is unlikely to change the broader and arguably, entrenched view, that vaginal breech birth is unsafe. Can you provide suggestions for how this somewhat systemic view of breech birth might be challenged?

Are the rationale for, and objectives of, the Systematic Review clearly stated?

Yes

Is the statistical analysis and its interpretation appropriate?

Not applicable

Are sufficient details of the methods and analysis provided to allow replication by others?

Yes

Are the conclusions drawn adequately supported by the results presented in the review?

Yes

Reviewer Expertise:

Midwifery, maternity care, women's experiences of maternity care, informed decision making

I confirm that I have read this submission and believe that I have an appropriate level of expertise to confirm that it is of an acceptable scientific standard.

# Reviewer report: Reviewer response for version 1

**DOI:** 10.3310/nihropenres.14455.r29778

**Published:** 2023-07-20

**Authors:** Martine H. Hollander

**Affiliations:** 1 Department of Obstetrics and Gynaecology, Radboud University Medical Center, Nijmegen, The Netherlands

Thank you to the editor and authors for allowing me to review this very interesting article, the subject of which is very close to my heart. I have some minor points for the authors’ consideration.

**Abstract:**

1. How can one seek an unplanned breech birth?

**Methods:**

1. Can the authors explain why they chose such a wide interval for their studies? January 2000 was before the publication of the Term Breech Trial, but no doubt many other things in the management of breech position have changed in the intervening years.
2. Figure 2, the concept map, shows the themes found, and therefore belongs in the results section.
3. I am missing a description of the background of the authors/researchers. In qualitative studies, the eyes looking at the data are never neutral, that would be impossible. So it would help to know the positions of the authors and the level of their involvement in breech care.

**Results:**

1. Very well written, interesting quotes and easy to glean the larger picture.
2. Figure 3 is interesting and novel! Has this been done before? Do you have a reference for this?

**Discussion:**

1. I am missing a wider scope for this issue. Women encounter physicians who are inexperienced and/or afraid. The causes of this would be interesting. The Term Breech Trial, the reduced experiences for trainees, the increase in audits and legal ramifications…
2. A parallel could be drawn with the literature on birth choices against medical advice. Those women also encounter a lack of autonomy, and report more satisfaction when they have felt heard, regardless of how the actual birth went.
3. Some comment could be made on the different setting and approaches to breech birth, especially in light of the tone on international breech platforms on social media. In many countries, VBB is a regular option, even if not exactly welcomed by all providers. However, in the US, this is mostly not the case. This has led to a whole subculture of service users (or rather, refusers) on the internet who seem to want to convince all women with a breech presentation that it is no different than vertex, and that a home birth is safe.
4. Do the authors have any concrete suggestions for dedicated services? And about how they would impact unexpected breech births and twins births?

Are the rationale for, and objectives of, the Systematic Review clearly stated?

Yes

Is the statistical analysis and its interpretation appropriate?

Not applicable

Are sufficient details of the methods and analysis provided to allow replication by others?

Yes

Are the conclusions drawn adequately supported by the results presented in the review?

Yes

Reviewer Expertise:

Maternity care

I confirm that I have read this submission and believe that I have an appropriate level of expertise to confirm that it is of an acceptable scientific standard.
